# Supplementary material for: Notch1 signaling determines the plasticity and function of fibroblasts in diabetic wounds
Source: Life Sci Alliance. 2020 Oct 27;3(12):e202000769. doi: 10.26508/lsa.202000769 (PMC7652398; doi:10.26508/lsa.202000769)
Supplement: Supplementary file 1 [file LSA_LSA-2020-00769_TableS1.docx]

Table S1.

| Sample | Age | Gender (M/F) | Ethnicity  (AA/A/H/HW/W) | HbA1c |
| --- | --- | --- | --- | --- |
| **DFUF** | 68 | M | W | 6.6 |
| **DFUF** | 60 | M | HW | 6.0 |
| **DFUF** | 68 | M | W | 8.1 |
| **DFUF** | 38 | M | AA | 7.3 |
| **DFUF** | 63 | M | W | 6.5 |
| **DFUF** | 79 | M | W | 6.6 |
| **NFF** | 55 | F | H | non DM |
| **NFF** | 62 | F | HW | non DM |
| **NFF** | 67 | F | HW | non DM |

Patient demographics and sample information. AA = African-American, H = Hispanic, HW = Hispanic White, W = White. DFUF = diabetic foot ulcer fibroblasts NFF=normal foot fibroblasts. HbA1c=Hemoglobin A1c. DM = diabetes mellitus.
